# Deep learning methods for high-resolution microscale light field image reconstruction: a survey

**DOI:** 10.3389/fbioe.2024.1500270

**Published:** 2024-11-18

**Authors:** Bingzhi Lin, Yuan Tian, Yue Zhang, Zhijing Zhu, Depeng Wang

**Affiliations:** ^1^ College of Energy and Power Engineering, Nanjing University of Aeronautics and Astronautics, Nanjing, China; ^2^ Department of Biomedical Engineering, Duke University, Durham, NC, United States; ^3^ Key Laboratory of Novel Targets and Drug Study for Neural Repair of Zhejiang Province, School of Medicine, Hangzhou City University, Hangzhou, China

**Keywords:** deep learning, light field microscopy, light field imaging, high resolution, volumetric reconstruction

## Abstract

Deep learning is progressively emerging as a vital tool for image reconstruction in light field microscopy. The present review provides a comprehensive examination of the latest advancements in light field image reconstruction techniques based on deep learning algorithms. First, the review briefly introduced the concept of light field and deep learning techniques. Following that, the application of deep learning in light field image reconstruction was discussed. Subsequently, we classified deep learning-based light field microscopy reconstruction algorithms into three types based on the contribution of deep learning, including fully deep learning-based method, deep learning enhanced raw light field image with numerical inversion volumetric reconstruction, and numerical inversion volumetric reconstruction with deep learning enhanced resolution, and comprehensively analyzed the features of each approach. Finally, we discussed several challenges, including deep neural approaches for increasing the accuracy of light field microscopy to predict temporal information, methods for obtaining light field training data, strategies for data enhancement using existing data, and the interpretability of deep neural networks.

## 1 Introduction

By simultaneously capturing combined signals from different depths of an entire volume in a single-camera-frame, light field microscopy (LFM) enables rapid spatial dynamic imaging (Levoy et al., 2006), and has developed into a valuable tool for structural and functional imaging of biological specimens. LFM usually necessitates computational volumetric reconstruction using traditional algorithms like refocusing (Dansereau et al., 2015; Jayaweera et al., 2020) or three-dimensional (3D) deconvolution (Broxton et al., 2013). However, conventional algorithms are limited by low efficiency and poor resolution, thereby hindering them for broader application of LFM. Therefore, the need to achieve high efficiency and high-resolution image reconstruction is crucial for the advance of LFM.

In recent years, deep learning has been widely used for variant applications, including image classification (Yu et al., 2022; Dosovitskiy, 2020; Foret et al., 2020), semantic segmentation (Wang et al., 2023; Srivastava and Sharma, 2024; Erisen, 2024), generation (Kim et al., 2023; Walton et al., 2022; Sadat et al., 2023), denoising (Zhou et al., 2020), restoration (Wan et al., 2022), super-resolution (Johnson et al., 2016; Ledig et al., 2017; Shi et al., 2016), depth estimation (Alhashim, 2018; Zhuang et al., 2022; Tateno et al., 2018) and image reconstruction (Drozdova et al., 2024; Zhang et al., 2024; Godard et al., 2017; Li et al., 2017; Liang et al., 2021; Quan et al., 2021; Schlemper et al., 2017). The use of deep learning algorithms has also boosted LFM (LeCun et al., 2015; Vizcaino et al., 2021a; Wang et al., 2021; Yi et al., 2023; Wagner et al., 2021). For instance, deep learning-based LFM has been applied to resolve the activity of motor neurons in *Caenorhabditis elegans* with single-cell resolution (Wang et al., 2021), to extract the calcium signal in the brains of 5-day-old transgenic zebrafish (*Danio rerio*) larvae (Wagner et al., 2021), and to reconstruct the high-speed 3D voltage imaging in sparsely labeled dopaminergic neurons in the fruit fly brain (Lu et al., 2023).

This review first introduces light field acquisition methods, and explores the applicability of these methods in light field microimaging, then introduces the current application of deep learning in image processing technology and explores the feasibility of deep learning technology in light field microimaging reconstruction, and finally outlines the recent progress of deep learning-based reconstruction algorithms for LFM. This paper aims to provide a comprehensive review of deep learning-based LFM, focusing primarily on network architecture, reconstruction resolution, and running time to reveal current shortcomings, and future possibilities.

## 2 Light field and deep learning

### 2.1 Principle of light field imaging

In the field of 3D space, the light field serves as a comprehensive representation of all light rays existing in 3D space from any given point in any direction. The concept of the “Light Field” was first introduced by Alexander Gershun (Gershun, 1939), who proposed five-dimensional (5D) plenoptic function $L\left(x,y,z,\theta ,\varphi \right)\in R^{5}$ to describe the light field, utilizing spatial coordinates $\left(x,y,z\right)$ and angular coordinates $\left(\theta ,\varphi \right)$ to specify each ray. In contrast, Levoy and Hanrahan (Levoy and Hanrahan, 2023) presented four-dimensional (4D) representation $L\left(u,v,s,t\right)\in R^{4}$ for the light field, conceptualizing it as comprised of oriented lines in free space. This representation is efficient in reducing data redundancy and simplifying the reconstruction of the plenoptic function. The parameterization $L\left(u,v,s,t\right)$ defines these lines based on their interactions with two arbitrarily positioned planes, where the first plane is denoted by $\left(u,v\right)$ and the second plane by $\left(s,t\right)$ . All light field microscopy discussed subsequently in this paper is 4D light field.

**FIGURE 1:**
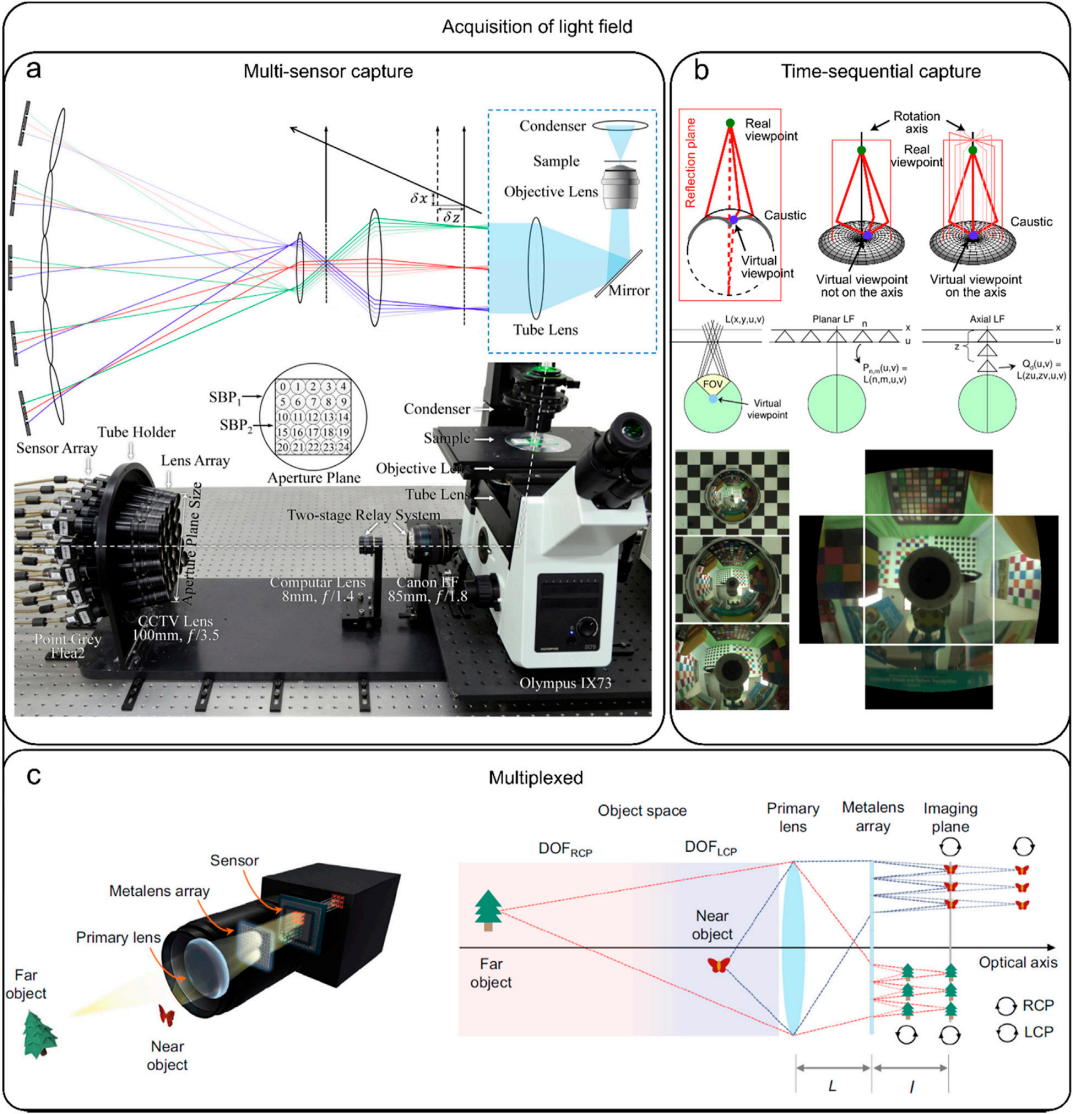
Acquisition of light field. **(A)** Multi-sensor capture: capturing the light field simultaneously using multiple cameras, most of which are camera arrays. [modified from (Lin et al., 2015)] **(B)** Time-sequential capture: capturing the light field using multiple exposures from a single camera, which is time-consuming. [modified from (Taguchi et al., 2010)] **(C)** Multiplexed capture: The process of mapping complex high-dimensional data into two-dimensional (2D) images. [modified from (Fan et al., 2022)].

Typically, there are three strategies to acquire light field information (Wu et al., 2017), multi-sensor capture (Figure 1A), time-sequential capture (Figure 1B), and multiplexed imaging (Figure 1C). Theoretically, all these three strategies aim to acquire light field information but the approaches they utilize to record light field information are totally different. Specifically, multi-sensor capture utilizes multiple cameras to concurrently capture light field, predominantly employing camera arrays (Lin et al., 2015; Huang et al., 2023; Gu et al., 2020; Xu et al., 2020). This approach can yield high spatial resolution imaging while capturing real-time information, but the total setup is complex and expensive. In time-sequential capture, a single camera is utilized to capture light field through a series of exposures. This method is known for being time-consuming and cannot provide real-time information (Li et al., 2014; Taguchi et al., 2010; Dansereau et al., 2017; Liu et al., 2022). On the other hand, multiplexed imaging involves the conversion of high-dimensional data into a more simplified two-dimensional (2D) image (Prevedel et al., 2014; Mignard-Debise, 2015; Kim et al., 2016; Vizcaino et al., 2021b; Orth and Crozier, 2012; Yang and Yuste, 2017; Fan et al., 2022) using a microlens array (MLA) positioned in the optical instrument’s intermediate image plane. By adopting this approach, the entire imaging system is significantly streamlined with easy operation. Consequently, light field camera and LFM developed based on the multiplexing principle are widely used in volumetric imaging. Particularly, LFM has demonstrated strong imaging ability in *in-vivo* imaging of heartbeat, blood flow, and neural activity, and has allowed 3D visualization of the spatial and temporal evolution patterns of the signals and the mechanisms behind biological processes.

### 2.2 Light field image reconstruction benefits from deep learning

Light field reconstruction can be seen as a transformation between the raw light field image and the reconstructed volumetric image. Classical reconstruction methods address such transformations from a physical-optical perspective through display modeling, which can be classified into two categories: mathematical inversion and numerical inversion. Refocusing is a typical mathematical inversion method in light field reconstruction (Ng et al., 2005; Alain et al., 2019) and is based on an idealized mathematical model, that essentially superimposes and shifts sub-aperture images over the entire aperture range. During the reconstruction process, the difference between the actual situation and the mathematical model is magnified, and thus prone to image noises, and artifacts. Numerical inversion employs iterative reconstruction for various imaging modalities to introduce external *a priori* information, thereby greatly enriching the information available for reconstruction and improving the quality of the final image. One widely used numerical inversion method is Richardson-Lucy deconvolution which relies on the microscope’s point spread function and Poisson noise statistics assumption (Prevedel et al., 2014; Richardson, 1972). However, the accuracy of these classical reconstruction methods is restricted by the premises of their physical models. These methods are unable to capture the full statistical complexity of microscopic images, and thus can only reconstruct high-quality results in specific cases. In contrast, data-driven procedures, especially deep learning methods, rely on high-resolution data to optimize the reconstruction procedure, thereby usually offering better resolution than conventional algorithms. Consequently, deep learning-based methods allow high-resolution light field reconstruction. For instance, deep learning methods have enabled high-resolution LFM in the reconstruction of fluorescently labeled blood vessels in mouse brain slices (Vizcaino et al., 2021a), neuronal signals and analysis of the calcium activity patterns, four-dimensional dynamics of red blood cells and cardiomyocytes (Wang et al., 2021), continuous 3D observation of dynamic processes (Yi et al., 2023), and imaging of zebrafish (*Oryzias latipes*) embryos and zebrafish (*D. rerio*) larvae (Wagner et al., 2021).

**FIGURE 2:**
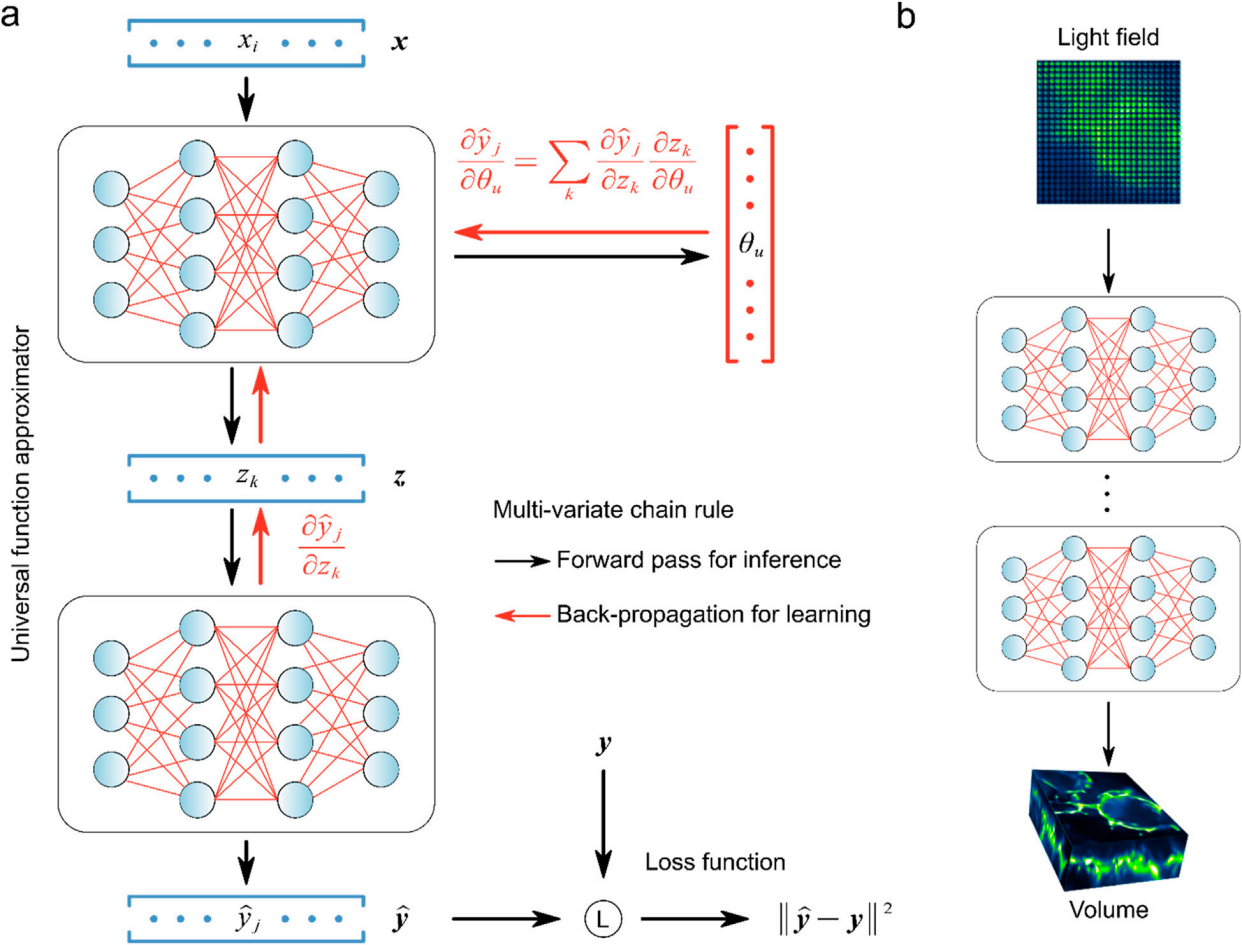
Universal function approximator and back-propagation were used to learn light field reconstruction. **(A)** Learning process of universal function approximator. **(B)** Multilayer neural networks and back-propagation were used to learn LFM direct reconstruction. [modified from (Lu et al., 2023)].

Briefly, deep learning networks are primarily composed of various nonlinear parameterized processing modules that iteratively convert an input x into the anticipated output y , generally approximating it as $\hat{y}$ . It is theoretically posited that a neural network possessing an ample quantity of parameters and a minimum of three layers can approximate virtually any function within its domain (Hornik et al., 1989). This assertion is founded on the universal approximation theorem in the field of networks, which suggests that with a sufficiently complex architecture, including an adequate number of parameters and layers, the network can flexibly adapt and represent a vast array of intricate functions. Such a network structure enables the model to intricately capture the underlying patterns and relationships present in the data, facilitating the acquisition of high-level abstraction to effectively model complex functions, and thus network can also be called a universal function approximator (Figure 2A). The main principle of learning is to make accurate updates to the parameter values θ . The learning process has two main steps. The first step requires passing the input value x of the module forward once in the network to get the approximation value $\hat{y}$ . The differentiable nature of each module within a neural network allows for the computation of partial derivatives for the parameters θ . This property enables the determination of how changes in these parameters impact both the output $\hat{y}$ and intermediate values z throughout the network architecture. By calculating the partial derivatives $\frac{\partial \hat{y}}{\partial \theta }$ and $\frac{\partial z}{\partial \theta }$ , it becomes feasible to assess the sensitivity of the network’s predictions and internal representations to variations in the model’s parameters. This differentiation capability plays a fundamental role in the optimization process, as it facilitates the adjustment of parameter values to minimize the discrepancy between the network outputs and the anticipated output. Ultimately, the ability to compute these partial derivatives for the parameters empowers the neural network to iteratively refine its internal representations and enhance its predictive performance through the optimization of its parameters. Therefore, the second step utilizes the back-propagation algorithm (Rumelhart et al., 1986) to iteratively update the initially set θ value by efficiently calculating all the partial derivatives or gradients by the chain rule and passing them backward once. Using the loss function $l\left(y,\hat{y}\right)$ , the difference between the anticipated output y and the network output $\hat{y}$ can be measured. To minimize this loss, a commonly employed approach involves the utilization of optimizers such as Adam (Kinga and Adam, 2014) to adjust the parameter denoted as θ iteratively.

The trained network is the solver to compute volumetric images from the raw light field data, and directly impacts the quality of the reconstructed image. Typically, to ensure the network is fully optimized, high-resolution 3D images of the target samples are first acquired, which can be obtained from simulated data or experimental methods, such as confocal microscopy (Vizcaino et al., 2021a), selective plane illumination microscopy (SPIM) (Wagner et al., 2021) and light-sheet microscopy (Zhao et al., 2020). Using the wave optics model, these high-resolution volumetric images are projected into 2D light field images. In the network training process, the raw light field images serve as the initial input denoted as x . The network then extracts relevant features by image convolution with kernels, ultimately generating a set of 3D image stacks represented as $\hat{y}$ (Figure 2B). Lastly, a suitable loss function must be chosen. The loss function is then minimized and kernels are updated using the network iteratively. This process continues until the network is gradually optimized to the point where it can produce a 3D image that closely resembles the ground truth from the synthetic light field.

## 3 Deep learning-based reconstruction algorithms for light field microscopy

**FIGURE 3:**
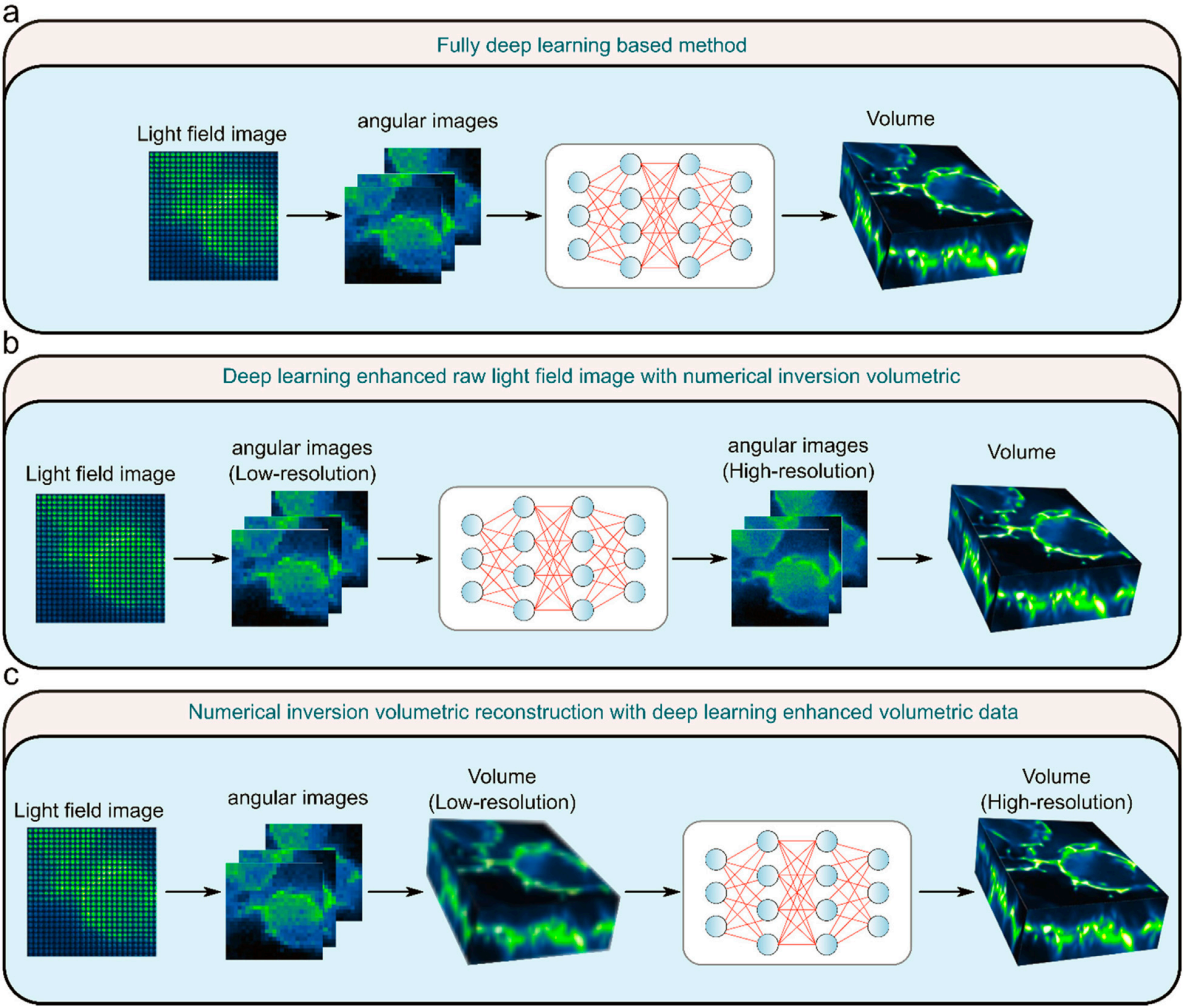
Three types of light field microscopy reconstruction methods. **(A)** Fully deep learning-based method. **(B)** Deep learning enhanced raw light field image with numerical inversion volumetric. **(C)** Numerical inversion volumetric reconstruction with deep learning enhanced volumetric data. [modified from (Lu et al., 2023)].

Deep learning-based LFM image reconstruction has demonstrated superior resolution than conventional methods (Wang et al., 2021; Yi et al., 2023; Wagner et al., 2021), which allows researchers to observe finer structures, such as subcellular organelles or molecular complexes with greater clarity. The improved performance of deep learning-based methods originates from its upsampling design in the network, which can compensate for the reduced resolution of raw light field images when the volumetric information is encoded onto the 2D sensor. To attain high resolution and high efficiency, deep learning methods can also be integrated with numerical inversion strategy. Based on the combination of deep learning and numerical inversion methods, the current deep learning-based LFM algorithms can be subdivided into three categories: fully deep learning-based method (type I) (Figure 3A), deep learning enhanced raw light field image with numerical inversion volumetric reconstruction (type II) (Figure 3B), and numerical inversion volumetric reconstruction with deep learning enhanced volumetric data (type III) (Figure 3C).

Type I method completely uses deep learning to reconstruct the raw light field image into a 3D volume. This network needs to accomplish both volumetric reconstruction and resolution improvement tasks simultaneously. The Type II method initially uses deep learning algorithms to elevate the resolution of the raw light field image, succeeded by a gradual reconstruction of the light field image through the utilization of numerical inversion methods. Type III method refers to the use of the numerical inversion method to iteratively reconstruct the light field image into poor 3D volume, and then use the deep learning method to transform the low-resolution volume into high-resolution volume. Specifically, the performance of these three types of methods varies depending on the structure of the variant. Type I methods use an end-to-end network, which has the advantage of being able to quickly reconstruct a volumetric image from a light field image as long as the network is appropriately trained, but is more difficult to train due to the complexity of the network. Compared to Type I methods, Type II and Type III methods have the advantage of better generalization, but the numerical inverse volumetric reconstruction in them requires iterative computation, resulting in less efficient reconstruction. The Type III method has a wider range of applications than the Type I and Type II methods, but the drawback is that false results that deviate too much from the real situation may occur. Each of these three types of methods has its own advantages and disadvantages, which need to be considered and weighed when applying them. In the future, type I methods may become the mainstream of real-time reconstruction of LFM, type II methods may become the mainstream of high-resolution reconstruction of LFM, and type III methods will be applied to a variety of 3D reconstruction in addition to LFM.

### 3.1 Type I: Fully deep learning-based method

**FIGURE 4:**
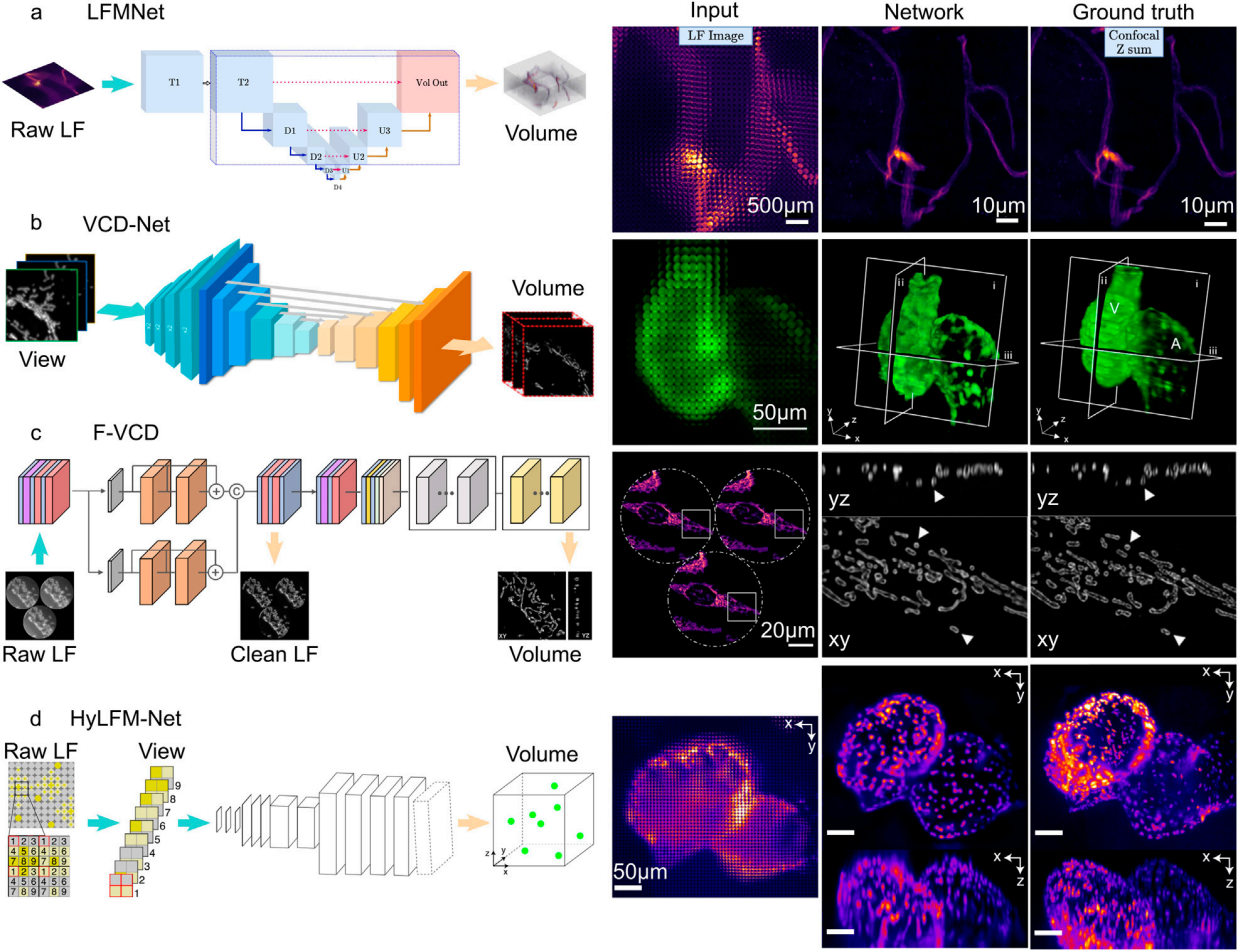
Fully deep learning-based method of light field microscopy. **(A)** LFMNet architecture (left), and the imaging result (right). Scale bars, 500 μm (LF image), 10 μm (Network and Ground truth). [modified from (Vizcaino et al., 2021a)] **(B)** VCD-Net architecture (left) and the imaging result (right). Scale bars, 50 μm . [modified from (Wang et al., 2021)] **(C)** F-VCD architecture (left), and the imaging result (right). Scale bars, 20 μm . [modified from (Yi et al., 2023)] **(D)** HyLFM-Net architecture (left) and the imaging result (right). Scale bars, 50 μm . [modified from (Wagner et al., 2021)].

The fully deep learning-based method is the most commonly used deep learning-based method for light field reconstruction (Figure 4). This approach uses the light field image as the input x for conventional deep learning, generating the predicted volume as the output $\hat{y}$ , while the target volume (ground truth) serves as the desired output y (Figure 2B). The most advanced networks based on fully deep learning-based methods currently include LFMNet, VCD-Net, F-VCD, and HyLFM-Net (Figures 4A–D).

In these networks, LFMNet (Vizcaino et al., 2021a) is the earliest architecture, which adds an initial layer (Conv4d (Choy et al., 2019)) to the U-Net. This design produced a fully convolutional network with the first layer traversing each microlens and capturing its surrounding neighborhood. The resulting output is then transformed into a channel number that is equal to the depths that need to be reconstructed. Subsequently, the tensor enters the U-Net for feature extraction and 3D reconstruction. The LFMNet has been mainly validated on images of fluorescently labeled blood vessels in mouse brain slices and achieved reconstruction resolution $\left(0.086\;\mu m\right)$ that was comparable to confocal microscopy. Compared to previous methods, LFMNet has significantly improved reconstruction accuracy, such as Peak Signal-to-Noise Ratio (PSNR) and Structural Similarity (SSIM), and reconstruction speed (75,000 times faster than deconvolution).

Following LFMNet, VCD-Net (Wang et al., 2021) was proposed, which adopts the cascaded convolutional layer design of the U-Net architecture, but differs from LFMNet in that the initial layer is no longer designed using Conv4d. Instead, the initial layer is transformed using SubPixel up-scaling (Shi et al., 2016) and a convolutional layer to reformat pixels in the input 2D light field raw image into different views, generating multi-channel outputs representing different depths. VCD-Net has performed single-cell resolution and up to 200 Hz volumetric imaging on the neuronal activity of moving *C. elegans* and the blood flow of beating zebrafish hearts, and has obtained uniform average resolutions within the range of the 1.1 μm in both the axial directions.

F-VCD (Yi et al., 2023) is proposed based on VCD-Net, so it provides improved reconstruction resolution, accuracy, and efficiency over VCD-Net. The F-VCD comprises two primary modules: the “F-Denoise” module and the “F-Reconstruction” module. The F-Denoise module introduces a viewing angle attention branch into the traditional RCAN network (Chen et al., 2021) to balance the influence of different viewing angles, to denoise raw light field images in a weighted way, because light field images from different viewing angles have different signal-to-noise ratio (SNR). The F-Reconstruction module is based on VCD-Net but has added three dilated convolution blocks to the original U-Net coding blocks of VCD and replaced the normal convolution operation with a residual block. This increases the number of input channels and expands the lateral size of the extracted features. To prevent the loss of subtle signals in the optimization process of deep networks, adjustments have been made to the normalization layer and activation function, replacing them with instance normalization and LeakyRelu, respectively. The F-VCD has been mainly validated on live-cell imaging and fixed-cell imaging. In live-cell imaging, the F-VCD technique enabled the achievement of 3D super-resolution imaging with a resolution of approximately 180 nm × 180 nm × 400 nm and was able to capture the rapid motion and morphological changes of mitochondria within cells, including mitochondrial fusion, fission, and dynamic tubulation, at a rate of up to 50 Hz. In fixed-cell imaging, F-VCD significantly improved the spatial resolution and contrast, and reduced axial artifacts, enabling clear visualization of organelle structures such as mitochondria and the endoplasmic reticulum. Specifically, F-VCD improved the axial resolution from approximately 400 nm to approximately 320 nm and achieved a 2-fold increase in lateral and a 1.5-fold increase in axial resolution.

HyLFM-Net is different from the above methods. Instead of using a U-Net, HyLFM-Net (Wagner et al., 2021) consists of a series of residual blocks (Kaiming et al., 2016) and transposed convolutions. It converts the multi-channel 2D image to the axial spatial dimension after applying 2D residual blocks and transposed convolutions, resulting in a 3D image. This 3D image undergoes further processing through 3D residual blocks and is upsampled by transposed convolutions to ultimately obtain the reconstructed 3D volume. In the dynamic imaging of the 8-day-old zebrafish (*O. latipes*) embryonic heart, HyLFM-Net successfully imaged the dynamic of the zebrafish heart within a field of view of 350 × 300 × 150 $\mu m^{3}$ at a volume imaging speed of 40–100 Hz, with significant improvements in spatial resolution and image quality, and can achieve a 3D volume inference speed of 26.7 Hz on a consumer-grade GPU, at least 1,000 times faster than conventional LFD.

The utilization of entirely deep learning-based approaches holds the potential to significantly reduce the presence of mosaic-like artifacts in the vicinity of the focal plane, a prevalent occurrence in LFD (Prevedel et al., 2014) and can accurately recover the signal even when the SNR of the raw image is low (Vizcaino et al., 2021a; Wang et al., 2021; Yi et al., 2023; Wagner et al., 2021). However, this approach has some drawbacks because the network simultaneously improves the resolution and spatial-angular of light field, which may increase the training workload and lead to structure missing and image artifacts. To improve this problem, it is necessary to improve the adaptability of the network structure and loss function to achieve satisfactory prediction results.

### 3.2 Type II: Deep learning enhanced raw light field image with numerical inversion volumetric reconstruction

Scanning LFM (sLFM) system (Morozov et al., 2002; Wu et al., 2021) improves the raw light field image quality through physical scanning to collect the 4D spatial-angular light distribution at near-diffraction-limited. However, sLFM usually requires a certain amount of time to scan the sample when acquiring the light field, and this spatial resolution improvement comes at the sacrifice of temporal resolution. To compensate for this deficiency, VsLFM (Lu et al., 2023) optimizes the scanning process using a deep learning model based on DAOSLIMIT (Wu et al., 2021).

**FIGURE 5:**
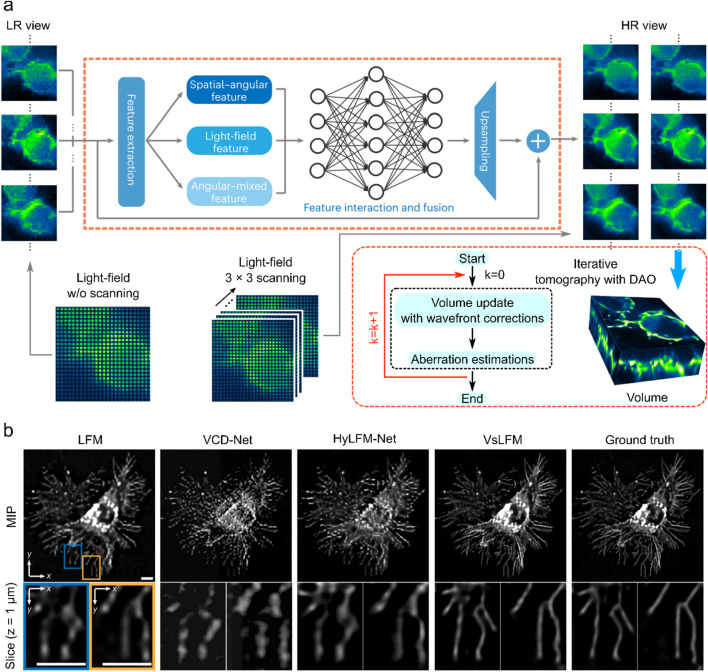
Deep learning enhanced raw light field image with numerical inversion volumetric reconstruction. **(A)** VsLFM schematic diagram. **(B)** Enhanced sections extracted from xy cross-sections at z = 1 μm of a static featuring membrane marking, captured using LFM, VCD-Net, HyLFM-Net, VsLFM, and sLFM techniques individually. Scale bars, 10 μm . [a and b modified from (Lu et al., 2023)].

After the light field’s resolution is increased, VsLFM is a typical network that is used for reconstruction. This process primarily uses deep learning to improve the raw light field image’s resolution. The subsequent reconstruction process necessitates the use of physical iterations to convert sample images from various angular views into volumetric images. To simulate the scanning process, VsLFM utilizes a supervised learning network (Vs-Net) to extract, interact, fuse and upsample spatial angle features. In network training, phase-dependent low-resolution angular data is used to learn physical priori relationships, and the high-resolution angular measurements produced by the sLFM are used as anticipated output. To rebuild 3D high-resolution volumes, iterative tomography is finally applied utilizing DAO on several angular views that are acquired by Vs-Net (Figure 5A). The subsequent reconstruction process requires the use of physical iterations to reconstruct the sample images from different viewpoints into volumetric images (Figure 5A). It is the improved resolution of the raw light field image that makes the imaging effect of VsLFM higher than that of the fully deep learning-based methods, especially in the localized details of specific depths where the image quality of VsLFM is close to ground truth.

VsLFM outperforms other methods such as LFM, VCD-Net, and HyLFM-Net on maximum intensity projection (MIP) images. VsLFM is able to obtain better resolution and contrast and performs well on both cell membrane-labeled and mitochondria-labeled samples (Figure 5B). In the numerical simulation of the synthesized 3D tubulins structure, the SNR of VsLFM in the spatial-angular domain is improved by about 15 dB and the SSIM is improved by 0.12. However, the increase in resolution involves physical iterations that can lead to time-consuming and poor reproducibility of the results. To address this issue, VsLFM has improved HyLFM-Net into HyLFM-A-Net to replace the physical iteration process with a deep neural network, which reduces the reconstruction of the whole process from 1,200 s to 11 s. The combination of Vs-Net and HyLFM-A-Net results in a deep neural network for mapping LF images to volumetric images. In contrast to the fully deep learning-based method, this approach is equivalent to tuning the network during the training process, so the complexity and redundancy of the model are much higher.

### 3.3 Type III: Numerical inversion volumetric reconstruction with deep learning enhanced volumetric data

**FIGURE 6:**
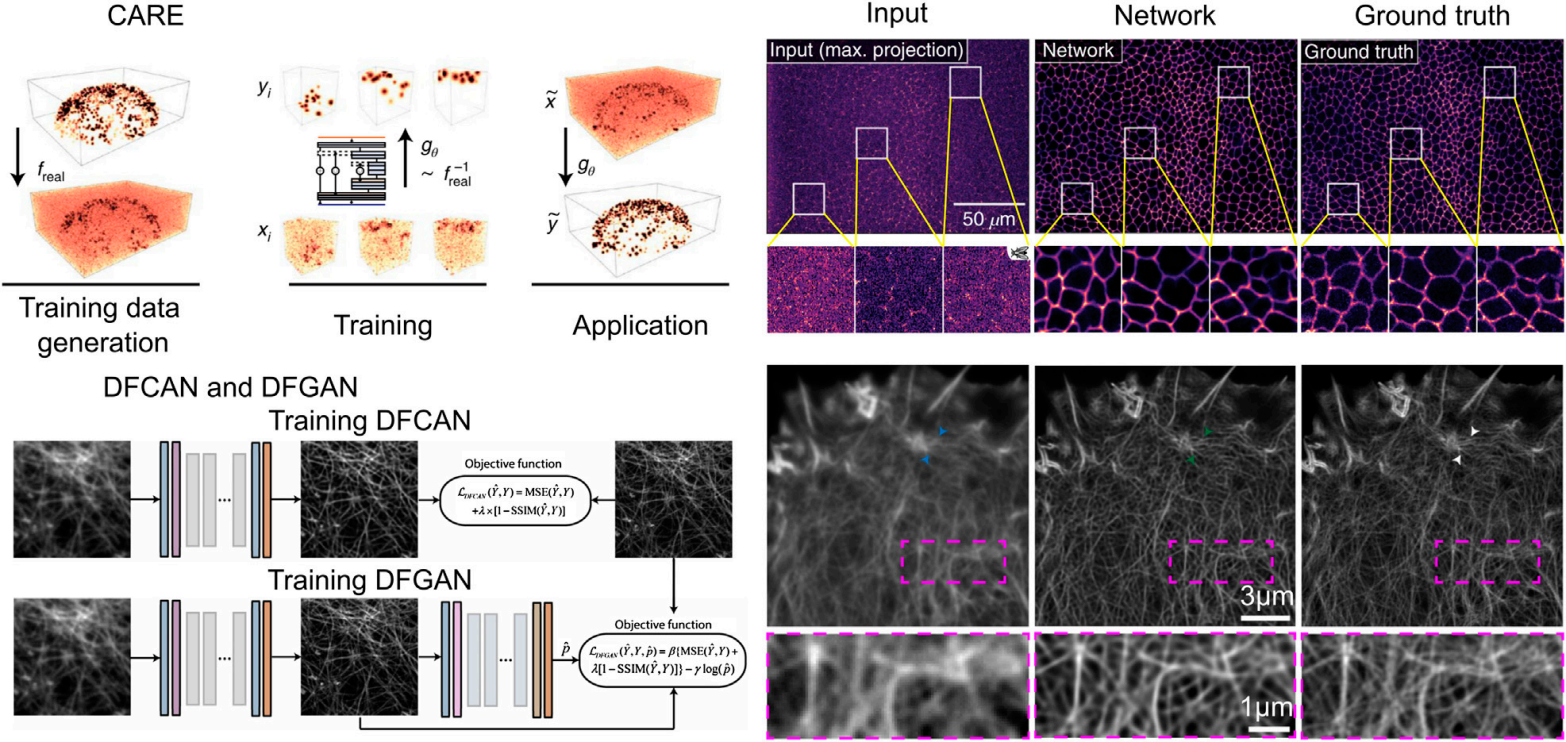
Numerical inversion volumetric reconstruction with deep learning enhanced volumetric data. **(A)** CARE architecture (left) and the imaging result (right). Scale bars, 50 μm . [modified from (Weigert et al., 2018)] **(B)** DFCAN and DFGAN architecture (left), and the imaging result (right). Scale bars, 3 μm (upper row), 1 μm (lower row). [modified from (Qiao et al., 2021)].

It is widely acknowledged that conventional numerical iterative algorithms are unable to produce satisfactory reconstructions due to the presence of redundancy in the majority of light field datasets. Consequently, researchers are faced with the challenge of fully utilizing the redundancy (Dansereau et al., 2015; Jayaweera et al., 2020; Mihara et al., 2016). Moreover, real optical physics has many deviations from the model so the corresponding errors propagate during the reconstruction process can cause image noise, blurring, and artifacts. To obtain higher resolution light field reconstruction, the results are usually further post-processed after reconstruction by conventional iterative methods. It is on this premise that the deep learning-based post-processing networks CARE (Weigert et al., 2018) (Figure 6A), DFGAN and DFCAN (Qiao et al., 2021) (Figure 6B), have also been applied to LFM.

CARE (Content-Aware Image Restoration) is a proposed method for LFM that utilizes machine learning techniques to enhance the quality of the acquired images. The primary objective of CARE is to develop a residual version of U-Net and train the network with a loss function mean square error (MSE). The CARE network can significantly improve the accuracy of cell nucleus segmentation with reduction in illumination dose, and has improved obvious segmentation accuracy (SEG) score from 0.47 in the original image to 0.65 in the CARE restored image. By leveraging machine-learned image computation, CARE networks can significantly improve image quality, making it easier to analyze biological samples.

After CARE was proposed, networks for single-image super-resolution (SISR) have also been proposed, and the most representative of these networks are DFCAN and DFGAN. DFCAN consists of convolutional layers, and DFCAN is a deeper DFCAN, which consists of convolutional layers, residual groups, Fourier channel attention blocks, skip connections, and activation functions such as GELU. The DFGAN network is derived from a conditional generative adversarial network (cGAN (Mirza, 2014)) framework applied to the DFCAN network. The generative model G of DFGAN is DFCAN, which mainly learns data distribution and image transformation. The discriminative model D is constructed based on the conventional CNN architecture, which consists of a convolutional layer activated by LeakyReLU and a fully connected layer activated by a sigmoid activation function. DFCAN and DFGAN validated the structures of clathrin-coated pits (CCPs), microtubules (MTs), and F-actin, and achieved good super-resolution reconstruction performance. Among them, in the case of 3-fold magnification, the quality of the reconstructed images is very close to the real super-resolution images, with the normalized root-mean-square error (NRMSE) below 0.1 on average. In addition, for the endoplasmic reticulum (ER) structure, due to the obvious aggregation caused by chemical fixation, the authors adopted real-time imaging and also obtained satisfactory reconstruction results.

This type of network can also be applied in other microscopy in fluorescent imaging, but it has certain drawbacks. For example, its performance could be compromised when handling samples with extremely complicated structures. In addition, widespread application in practical experiments may be limited by high-fidelity super-resolution information, especially when the network is applied in sample that contains structure absent from the training set. Moreover, this network is not ideal for intensity-based quantification, such as fluorescent substance counting, and cannot be used for all current image restoration challenges due to its nonlinear neural network prediction nature.

To better compare all the mentioned networks, we have summarized their structure and the performance of them in Table 1.

**TABLE 1:** Comparison of different networks.

Category	Network	Architecture	Metric	Running time
Full deep learning-based method	LFMNET (Vizcaino et al., 2021a)	Conv4d (Choy et al., 2019) And U-Net (Ounkomol et al., 2018)	Error with Confocal: 0.086 μm (test on vessels)	50 ms
	VCD-NET (Wang et al., 2021)	PixelShuffle (Shi et al., 2016) And U-Net (Ounkomol et al., 2018)	uniform average resolutions 1.1 μm (x,y) 3.0 μm (z) (test on isovolumetric subdiffraction fluorescent beads distributed in a hydrogel)	5 ms (test on *Caenorhabditis elegans* and the blood flow of beating zebrafish hearts)
	F-VCD (Yi et al., 2023)	RCAN (Chen et al., 2021) And VCD-NET (Wang et al., 2021)	Axial resolution than VCD-Net: 2-fold Lateral resolution: 1.5-fold	20 ms (test on mitochondrial)
	HyLFM-Net (Wagner et al., 2021)	2D residual blocks (Kaiming et al., 2016) And 3D residual blocks (Kaiming et al., 2016)	MS-SSIM 0.982 ± 0.002 (test on beads) 0.91 ± 0.02 (test on static) 0.78 ± 0.04 (test on dynamic) 0.90 ± 0.02 (test on brain)	10 ms (test on medaka heart dynamics and zebrafish neural activity)
Deep learning enhanced raw light field image with numerical inversion volumetric reconstruction	VsLFM (Lu et al., 2023)	convolutional layers Leakey ReLU And PixelShuffle (Shi et al., 2016)	spatial-angular domain SNR ∼30 dB SSIM ∼0.95 Reconstructed SNR ∼2.5 dB SSIM ∼0.6 (test on 3D tubulins structure)	1,200 s
Numerical inversion volumetric reconstruction with deep learning enhanced volumetric data	CARE (Weigert et al., 2018)	Residual version of U-Net (Kaiming et al., 2016; Ounkomol et al., 2018)	SEG score: from 0.47 to 0.65 (test on *Tribolium castaneum*)	
	DFCAN and DFGAN (Qiao et al., 2021)	Fourier channel attention (Chen et al., 2021), convolutional layers And PixelShuffle (Shi et al., 2016) (DFCAN and DFGAN) And cGAN (Mirza, 2014) (DFGAN)	NRMSE: 0.0593 MS-SSIM: 0.8665 Resolution: 139 nm (test on low-fluorescence average photon count 120 DFCAN) NRMSE:0.0586 MS-SSIM:0.8680 Resolution:97 nm (test on low-fluorescence average photon count 120 DFGAN)	

## 4 Challenges and opportunities

The existing reconstruction methods for LFM using deep learning are facing multiple challenges. Firstly, the current approaches are centered on predicting single frames, necessitating a refinement in ensuring accurate predictions for consecutive frames. Nonetheless, enhancing the forecast precision for continuous frames inevitably translates to an increase in computational burden, thereby escalating the requisites for advanced computational resources. Secondly, the scarcity of available LFM datasets poses a hindrance to fully harnessing these resources for achieving optimal outcomes. Lastly, the enigmatic nature of deep learning models presents a hurdle in enhancing the intelligibility of these intricate models.

### 4.1 Accuracy in predicting temporal information

Utilizing cutting-edge techniques in real-time data forecasting, researchers can accurately monitor the dynamic behaviors of numerous cells with precision in both the spatial and temporal domains, thereby enhancing comprehension of neuronal population activities (Prevedel et al., 2014; Hornik et al., 1989). Nonetheless, when applying deep learning methodologies to LFM, the conventional practice involves individual frame prediction, leading to potential inconsistencies in temporal coherence and the presence of artifacts over time intervals. To address this challenge effectively, it is crucial to explicitly account for temporal dynamics during data reconstruction by incorporating time-resolved data. While a straightforward approach involves treating time as an extra dimension within CNNs, such a method may not be viable for extensive networks managing prolonged correlations. An alternative and more efficient solution are to merge CNNs with advanced recurrent neural networks like convLSTM (Zhang and Zhang, 2024) and convGRU (Lagemann et al., 2021) architectures, which are specifically tailored for sequence prediction tasks. However, this integrated approach may demand more sophisticated hardware resources to ensure streamlined execution.

### 4.2 Hardware requirement

In the realm of deep learning for applications like LFM, the necessity for customized software frameworks to facilitate the manipulation and analysis of intricate neural networks is evident. A pivotal consideration in these advancements pertains to the evolving hardware prerequisites. As we all known, specialized graphics processors (GPUs) for training deep learning models underscores the criticality of hardware in expediting computational processes. The migration towards GPU utilization over conventional central processing units (CPUs) is essential for substantial gains in training speed, significantly reducing the training duration. This transition not only accelerates the pace of model refinement but also addresses the cost constraints associated with sophisticated hardware requirements. Looking ahead, the symbiotic relationship between software innovation and hardware optimization remains fundamental in shaping the trajectory of deep learning applications, paving the way for enhanced efficiencies and broader accessibility across research domains.

### 4.3 Better network structures and training strategies can reduce the need for datasets

Deep learning’s effectiveness is vitally dependent on the availability of training data. Inadequate training data will result in poor performance. However, a prevalent misperception is that deep learning requires an enormous amount of training samples. For example, VsLFM (Lu et al., 2023) training typically uses 5,000 paired spatial-angular patches, and VCD-Net (Wang et al., 2021) trains using 4,580 pairs of image patches, each with a light field image (176 × 176 pixels) and a volume (176 × 176 × 51 pixels). However, LFMNet (Vizcaino et al., 2021a) required 362 high-resolution images (1,287 × 1,287 pixels), whereas based on the U-Net architecture (Ounkomol et al., 2018) only used 40 images (1,500 × 1,500 pixels). It can be observed that different network architectures and training strategies can significantly reduce the size of the training dataset. The quality of the data and its relevance to the situation are likely more crucial. In order to advance further, LFM necessitates innovative experimental and computational approaches for the production of an increased quantity and quality of training data.

### 4.4 Strategies for obtaining a light field dataset and leveraging existing training data to enhance the dataset

Various strategies can be explored to acquire a comprehensive light field dataset. One avenue involves conducting specialized experiments tailored to capture the requisite images for training purposes. For instance, employing confocal microscopy and light field microscopy in tandem to capture pairs of high-quality volumetric images along with corresponding light field data from a stationary cell location can help validate LFM image reconstruction algorithm. Additionally, leveraging an in-depth understanding of the underlying physics governing light field propagation enables the utilization of forward model simulations to generate authentic images (Weigert et al., 2018; Nehme et al., 2018). Furthermore, the integration of neural networks presents a promising approach to dataset creation. Recent endeavors have focused on the development of cell generation models through adversarial generative techniques (Osokin et al., 2017; Goldsborough et al., 2017; Yuan et al., 2019), leading to the generation of synthetic images that can subsequently contribute to training reconstruction algorithms. Moreover, conventional approaches, such as data augmentation, present a feasible tactic for enriching datasets by creating diverse variations of existing images. This process involves employing methodologies like rotation, scaling, and manipulation of lighting conditions to expand the range of training samples available for model learning. An alternative efficacious approach involves the utilization of transfer learning (Zeiler and Fergus, 2014). By transferring knowledge from pre-trained models to new tasks, transfer learning enables the efficient utilization of learned features and representations, thereby enhancing the generalization capability and performance of the neural network on specific tasks. These techniques, rooted in the diversification of data and the strategic reuse of network knowledge, play pivotal roles in advancing the efficacy and adaptability of deep learning models across various domains and applications. By pretraining networks on extensive datasets sourced from different domains, transfer learning expedites convergence and enhances the generalization capabilities of the models (Weiss et al., 2016). This multifaceted approach holds significant promise for enriching the light field dataset and maximizing its efficacy in a research context.

### 4.5 Explainable/interpreting the deep neural network

The challenge of model interpretability emerges as a critical issue given the inherently opaque and enigmatic nature of deep neural networks (Zhang et al., 2018). To establish deep learning as a reliable component within LFM-based processes, it is important to explore the integration of conceptual frameworks and interactive graphical tools to elucidate the underlying rationale behind generating specific outcomes. Encouragingly, the field of computer vision has witnessed advancements in enhancing the interpretability of deep learning through various methodologies. These include delving into the essential components of input images for accurate predictions, scrutinizing the function of intermediate layers, analyzing the contributions of different module through ablation studies, constructing hierarchical explanatory graphs spanning across layers, and designing network architectures that prioritize interpretability. The adaptation of these techniques to the domain of light field imaging is deemed essential, calling for the development of specialized tools tailored to facilitate the interpretation of results. One potential avenue involves the creation of tools explicitly designed to explicate the rationale behind predicted outcomes, thereby fostering transparency and comprehension in the intricate mechanisms governing deep neural networks in LFM contexts.

### 4.6 Outlook for deep learning to microscale light field image reconstruction

Deep learning-based LFM is still in its nascent stage, but significant advancements have been achieved in leveraging deep learning techniques for this purpose. As we look forward, the future of deep learning-based LFM reconstruction may further highlight the utilization of expansive and high-quality big data sets to facilitate various forms of learning paradigms such as supervised, weakly supervised, self-supervised, or unsupervised learning. To promote broader adoption and enhancement of existing tools, as well as the development of novel ones, it is critical to create extensive datasets to meet the image analysis requirements of the broader life sciences data. These datasets should be publicly accessible, assisting skilled machine learning researchers to tackle biological challenges. It is evident that there are numerous unexplored applications awaiting discovery in this domain. It is hence advisable to simultaneously push forward the tool development and biological prediction processes, given that deep learning fundamentally thrives on data analysis.
